# Multi-Modal Convolutional Parameterisation Network for Guided Image Inverse Problems

**DOI:** 10.3390/jimaging10030069

**Published:** 2024-03-12

**Authors:** Mikolaj Czerkawski, Priti Upadhyay, Christopher Davison, Robert Atkinson, Craig Michie, Ivan Andonovic, Malcolm Macdonald, Javier Cardona, Christos Tachtatzis

**Affiliations:** 1 Department of Electronic and Electrical Engineering, University of Strathclyde, Glasgow G1 1XW, UKchristopher.davison@strath.ac.uk (C.D.); i.andonovic@strath.ac.uk (I.A.); malcolm.macdonald.102@strath.ac.uk (M.M.); christos.tachtatzis@strath.ac.uk (C.T.); 2 Department of Chemical Engineering, University of Strathclyde, Glasgow G1 1XJ, UK; j.cardona-amengual@strath.ac.uk

**Keywords:** image synthesis, internal learning, image inpainting, image super-resolution, multi-modal learning

## Abstract

There are several image inverse tasks, such as inpainting or super-resolution, which can be solved using deep internal learning, a paradigm that involves employing deep neural networks to find a solution by learning from the sample itself rather than a dataset. For example, Deep Image Prior is a technique based on fitting a convolutional neural network to output the known parts of the image (such as non-inpainted regions or a low-resolution version of the image). However, this approach is not well adjusted for samples composed of multiple modalities. In some domains, such as satellite image processing, accommodating multi-modal representations could be beneficial or even essential. In this work, Multi-Modal Convolutional Parameterisation Network (MCPN) is proposed, where a convolutional neural network approximates shared information between multiple modes by combining a core shared network with modality-specific head networks. The results demonstrate that these approaches can significantly outperform the single-mode adoption of a convolutional parameterisation network on guided image inverse problems of inpainting and super-resolution.

## 1. Introduction

The internal learning approach to computer vision is an alternate paradigm, where instead of learning to perform tasks based on an external set of samples, the information within the target image itself is used to solve image inverse problems such as inpainting or super-resolution. This has several benefits, but most importantly, it can reduce the effect of dataset bias and overfitting. Prior works have explored internal learning for image inverse problems [1,2], but tend to deal with single-modality data; most commonly, natural images. However, there are problems where synthesising data of spatially aligned images from multiple domains may be required. A prominent example of such a use case relates to the satellite image applications, where missing or corrupt regions in optical data can be imputed using the information from another source, such as Synthetic Aperture Radar (SAR) [3,4,5,6,7]. Similar challenges can be encountered in other domains, such as those involving image segmentation masks [8] or map-to-aerial pairs [8]. In these cases, internal learning techniques designed for a single domain are not architecturally optimised for handling multiple domains and may adapt poorly, yielding inferior performance. This motivates the technique proposed here, tailored for multi-modal image synthesis in an internal learning context.

**Figure 1:**
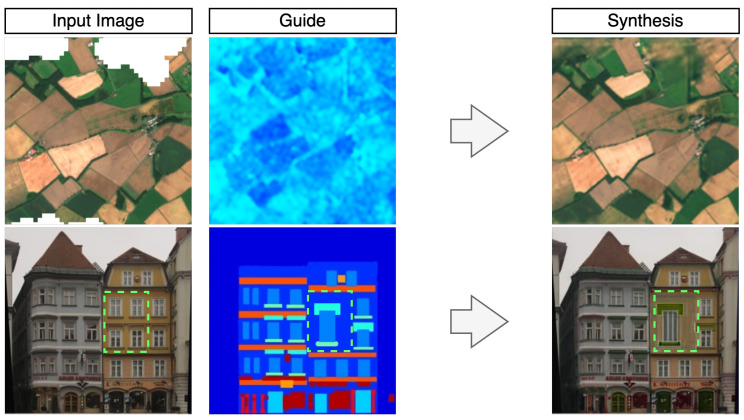
**Top row**: an example of using guidance information from Synthetic Aperture Radar (SAR) modality to inpaint an optical image. **Bottom row**: an example where a segmentation mask is used as a guide for manipulating an image for creative purposes; the synthesized region of the image is highlighted with a green dashed line (the rest of the image is learned in a supervised manner).

Already mentioned above, satellite images are a good example of the potential application of multi-modal image synthesis techniques and several relevant use cases are presented in this work. The utility of satellite images for Earth observation can be significantly reduced when portions of data are missing, which is a relatively common occurrence, especially in the case of optical sensors [9]. The gaps in data can be a result of sensor malfunctions or inherent limitations, such as cloud occlusion or shadows [9]. As a consequence, there is a need for methods that can manipulate satellite images to make them usable for downstream processing. The majority of existing deep learning solutions involve training on an external dataset with the aim of generalising new samples [10,11]. In recent years, several methods based on the internal learning paradigm have been proposed for satellite image manipulation tasks [12,13,14,15,16]. In these cases, the existing information in the source image may be more important than external domain knowledge, and hence, internal learning can avoid issues associated with transferability between regions. The methods proposed in [12,13,14] apply a convolutional module that transforms one signal modality into another, hence requiring an equal number of support and synthesised samples. Alternative solutions are proposed in [15,16] that stack multi-domain images on top of each other, allowing for any quantities of support and synthesised images to be used and effectively treat them as a single representation. This may produce distorted output caused by interference between channels from disparate domains. Here, a method is proposed to alleviate these issues by learning a representation of spatially aligned signals of multiple diverging domains, capable of solving inverse tasks of inpainting and super-resolution. This is done in a fully internal learning regime (no requirement of pretraining on an external dataset) by parameterising individual domain images with a convolutional neural network architecture, hereby named Multi-modal Convolutional Parameterisation Network (MCPN). Two use case examples of MCPN are shown in Figure 1, demonstrating how information from two drastically different domains can be shared to facilitate synthesis.

To demonstrate the capabilities of the proposed framework in a wider range of domains, additional use cases for a multi-modal convolutional parameterisation network are explored. Specifically, it is demonstrated how the datasets of spatially aligned images used in pix2pix [8] can also be subject to image completion. This includes two datasets, where natural images are paired with segmentation masks, one dataset with pairs of optical satellite imagery and corresponding map representation from Google Maps, and finally, pairs of natural images taken day and night. As shown in Figure 1, simplified representations, such as segmentation masks can be used for creative purposes to manipulate images. The map abstraction from Google Maps can be used as a useful static guide for cleaning up real aerial footage, for example, when clouds are present. Finally, the night-to-day task is performed for evaluation purposes to represent two diverging natural image domains.

The remainder of the manuscript is organized as follows. The MCPN method is detailed in Section 2, including a description of hyperparameter configuration, learning rate adjustment for the internal optimization process, and methods for extracting converged weights. That is followed by the evaluation of the methods in Section 3, which primarily focuses on the processing of satellite images, including both inpainting and super-resolution, as well as the evaluation of other common multi-domain image datasets, with the aim to highlight the potential applicability of MCPN to other problems. The conclusions are drawn in Section 4.

## 2. Method Description

**Figure 2:**
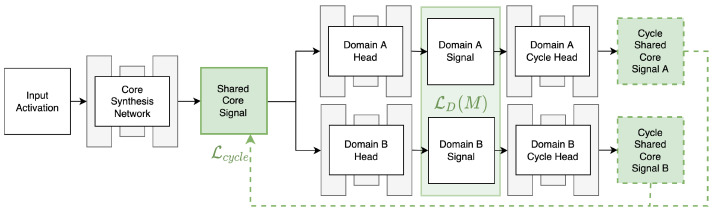
Diagram of the Multi-Modal Convolutional Parameterisation Network (MCPN). This example design can be readily adjusted for any number of domains. The arrows indicate the direction of the computation flow. The dashed arrows indicate that an additional loss term is produced as a comparison against what the arrow is pointing at.

The MCPN consists of a single core network for producing a shared signal representation, as shown in Figure 2. The core synthesis network is responsible for producing a shared core signal from which all domain-specific signals can be derived. The derivation is carried out by domain-specific convolutional heads that transform the shared core signal to individual target domains. In effect, spatial information sharing between the domains is enforced by relying on the same shared core signal. Finally, a set of domain cycle heads is used to convert each domain target signal back to the shared core signal and promote consistency of inpaintings. This arrangement yields two loss terms optimised by the network, the domain-specific loss $L_{D}\left(M\right)$ computed between the synthesised domain signals and the existing target reference, applied with an appropriate domain-specific mask *M*, and the cycle consistency loss $L_{cycle}$ computed as the difference between the shared core signal and the outputs of the cycle heads. Both losses $L_{D}\left(M\right)$, $L_{cycle}$ are computed as Mean Square Error (MSE) between the respective inputs.

**Figure 3:**
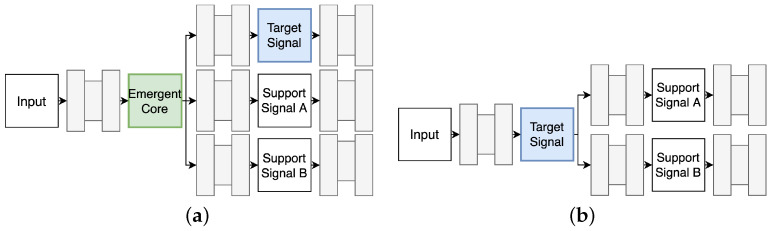
The Emergent Core variant (**a**) allows for a shared core signal to be learned in an emergent fashion, synthesising each domain signal, including the target image (blue) using specific heads. The Direct Core variant (**b**) instead uses the target image (blue) as the shared core signal, leading to one less domain head network.

The shared core signal is learned in an emergent fashion by backpropagating from the sum of individual domain-specific reconstruction losses $L_{D}\left(M\right)$ and the cyclic terms $L_{cycle}$. This variant of MCPN is referred to as Emergent Core MCPN and is further illustrated in Figure 3a. Another possibility is to use the synthesised signal of interest, such as an incomplete optical image, as the shared core representation, effectively dropping one of the domain-specific branches. Hence, $L_{D}\left(M\right)$ is defined as a sum of a loss directly computed on the synthesised signal of interest at the output of the core network, plus the domain-specific losses, at the output of the head networks. This variant is referred to as Direct CoreMCPN, as shown in Figure 3b.

### 2.1. Framework Configuration

**Figure 4:**
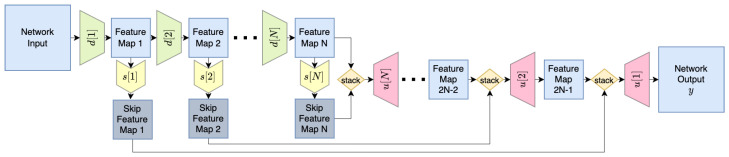
Architecture of the SkipNetwork, diagram based on Figure 21 from [2].

**Figure 5:**
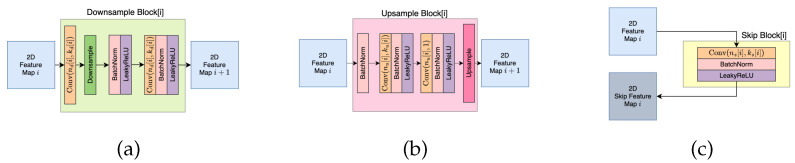
Three building blocks of a SkipNetwork: (**a**) a Downsample Block, (**b**) an Upsample Block, (**c**) an (optional) Skip Block.

The capacities of the core network and the domain-specific heads determine how much information is contained in the shared core signal. As the capacity of the domain-specific heads decreases, the possible transforms between the shared core and individual output images are simpler. This capacity can be controlled by the number of layers, their width, and the activation functions applied to the networks. In this study, the core network is identical to the SkipNetwork employed in the Deep Image Prior [2] (illustrated in Figure 4) for the inpainting task with the configuration where $n_{d}$ = $n_{u}$ = [16, 32, 64, 128, 128, 128] (where $n_{d}$ and $n_{u}$ are the numbers of channels of the downsampling and upsampling submodules, respectively) and skip modules of four channels. The domain-specific networks are also composed of similar elements, but contain only two stages of [32, 32] channels, along with skip modules also with [32, 32]. Submodules are illustrated in Figure 5. For the Emergent variant, downsampling and upsampling operations are maintained as in the Deep Image Prior reference network. For the Direct variant, a stride of 1 is used for all layers, resulting in a preserved representation shape.

Further important factors influencing this dynamic are the size of the convolutional kernels in the domain-specific heads and the number of channels of the shared core representation. If the domain-specific kernels are set to the size of 1 × 1, then all pixels of the shared representations are processed independently, which forces the shared representation to contain a lot of information for every output pixel. If the kernel size is increased, then the local neighbourhood information is passed on to the head network, meaning that individual shared pixels do not have to contain full global context information. In this study, based on exploratory analysis, it has been found that a core representation with eight channels for the Emergent variant, and head kernel sizes of 3 × 3 provide an appropriate baseline configuration. For the Direct variant, the number of channels in the core representation is by definition equal to the number of channels in the target representation.

In the evaluation section, and for all resulting images, the configuration listed in Table 1 is used for MCPN, unless otherwise stated. Optimisation is carried out by employing an Adam optimiser with standard parameter values. The learning rate and the number of optimisation steps are determined based on the convergence discussion in Section 2.2. Furthermore, the main baseline, similar to [16], that accommodates multiple nodes by expanding output channels and stacking the multiple representations on top of each other (hence, it is referred to as ‘Stacked‘ throughout the paper) only uses the core network, without any heads, with the exact same topology as the core network of MCPN.

### 2.2. Convergence Detection

Comparing convolutional parameterisation architectures, the proposed MCPN variants, and the Stacked baseline [16], is challenging because they may require different learning rates to allow stable convergence and a different number of optimisation steps. Hence, setting the same learning rate and applying the same number of weight updates for all architectures may put some of the models at a disadvantage and bias the evaluation. To explore this effect, a set of experiments is carried out where the performance computed based on the known ground truth is traced for 20,000 optimisation steps. The two metrics used to measure the quality of a synthesized image with reference to a specific ground truth are Structural Similarity Index (SSIM) and Root Mean Square Error (RMSE), similar to the related work in [16] (furthermore, both whole image metrics and inpainting mask metrics are reported). There are other metrics that focus on perceptual quality assessment, such as FSIM [17], IW-SSIM [18], TReS [19], or UNIQUE [20]; however, here, the focus is on diverse domains (such as satellite images), which do not always prioritise the human subjective scoring, and hence, more agnostic metrics of RMSE and SSIM are employed similarly to the previous work on the topic [16].

In the seminal work on the Deep Image Prior [2], the output was produced using the weights obtained after applying a fixed number of optimiser steps, depending on the task. In the related work that adapts the method for satellite image inpainting [16], 4000 steps were used.

Another approach is to devise an adaptive strategy for detecting a suitable convergence state. One solution for an adaptive convergence detection is to measure the performance of the synthesis on the known, non-masked region and find a stopping criterion on that quantity, for example, RMSE between the source image and the network output in the non-masked region. However, in some scenarios, the reconstruction error of the known region could monotonically decrease or saturate without ever reaching a minimum, while the error in the inpainting could be increasing, thus yielding a poor solution.

Here, an alternative adaptive approach that takes into consideration the inpainted region is proposed. This is conducted by measuring the quality of inpainting as the similarity of texture patches between the inpainted and known region of the image, termed the patch consistency metric. The metric is computed as the Fréchet distance between the two distributions of low-level features of a pre-trained Inception network [21] in response to the inpainting region and the distribution of features from the known region, in a similar fashion to Single Image Fréchet Inception Distance (SIFID) [22]. The metric requires the computation of a feature map $F_{source}$ of the source image and a feature map $F_{output}$ of the image produced by the network. Patch representations of the known and inpainted regions can be obtained by applying the inpainting mask to the $F_{output}$ and its inverse to $F_{source}$. Since the size of the feature maps is reduced in the layers of the Inception model, the mask *M* must also be reduced to apply it to the feature maps $F_{source}$ and $F_{output}$. This is achieved by downsampling the mask *M* in the same manner as the features to obtain a downsampled feature mask $M_{F}$. The feature mask $M_{F}$ equals 1.0 if and only if a given feature is affected by any pixel from the inpainting region. This way, the features extracted with the mask $M_{F}$ correspond to all features affected by the synthesised pixels, and the features extracted with the inverse mask $M_{F}^{\prime }$ filter out features that are only affected by the known region.

**Figure 6:**
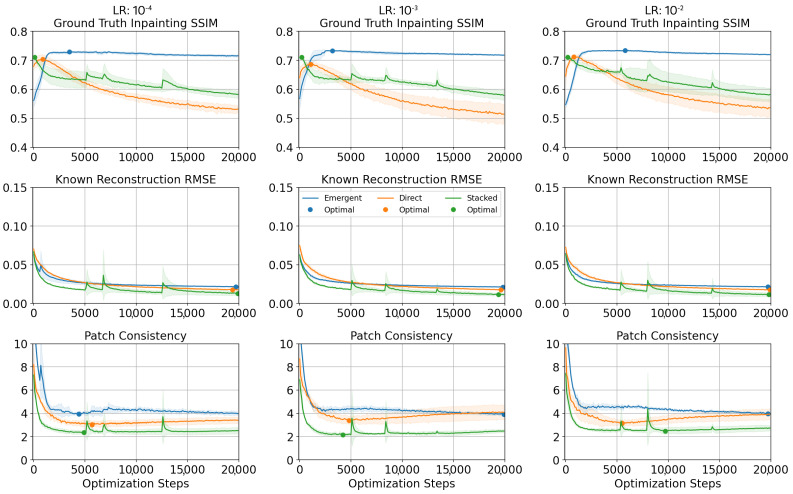
Convergence traces for a ground truth metric and two potential candidates for a stopping criterion.

Figure 6 demonstrates how the values of the two adaptive convergence metrics and ground truth inpainting SSIM evolve over the optimisation process. The rows in order correspond to (i) ground truth inpainting SSIM, (ii) known reconstruction RMSE, and (iii) patch consistency. The three columns correspond to the tested learning rates of $10^{-4}$, $10^{-3}$, and $10^{-2}$. The traces have been obtained for four repeated runs of inpainting a single Sentinel-2 sample based on Sentinel-1 informing data. The analysis of the convergence detection methods is performed for the SAR-to-optical synthesis because it represents the most challenging scenario where the gap between domains is considerable. The inpainting mask used covers the whole image except for a border of 50 pixels around the image periphery.

The top row in Figure 6 contains the traces recording the value of the inpainting SSIM, which has been computed with reference to the ground truth. The maximum value of the trace indicates the top performance that could be achieved by each model, but extracting it requires knowledge of the ground truth not available in practice. The maximum values of inpainting SSIM tend to occur within the first 5000 steps for all three model types, regardless of the learning rate (with the exception of Emergent Core at $10^{-2}$ learning rate). For the Stacked baseline (green line), this maximum value appears to be reached early in the process. This relates to the dynamics of Deep Image Prior convergence [2], where the low-frequency components are fit first before the fine detail. In effect, the low-frequency approximation at the beginning of the optimisation scores better than later solutions where high-frequency components are synthesised. The second and third rows contain the traces of the adaptive convergence detection metrics. The known region RMSE decreases monotonically (with occasional local spikes), and may be a poor choice for a proxy metric. In the third row, the patch consistency reaches minimum closer to the top performance states, but the two do not seem to align particularly well, for example, the minimum patch consistency is achieved long after the top performing SSIM for Direct and Stacked model variants.

As a supplement to Figure 6, Table 2 contains the average of extreme values of the inpainting SSIM, known region RMSE, and patch consistency for four repetitions. Furthermore, for each record, the table presents the mean and standard deviation of the number of optimisation steps after which the extreme value was reached. The Inpainting SSIM provides an indication of which learning rate results in the best performance for the tested sample. It is further apparent, that the minimised known region RMSE occurs very late in the training process and it is possible that it could keep converging further with more steps in the experiment. The patch consistency does reach a minimum value closer to the top performing Inpainting SSIM. Lastly, based on this result, the learning rate for each model has been selected, with $10^{-3}$ for MCPN Emergent and $10^{-2}$ for MCPN Direct and the Stacked baseline.

Using these learning rates, a broader experiment is conducted on the whole dataset of cloud-free images from Scotland (described in Section 3.1), with four repetitions on each sample, and with the same mask leaving out a 50-pixel border in the image. The average maximum inpainting SSIM that can be achieved is presented in the first column of Table 3, while the remaining columns contain the average inpainting SSIM obtained using the three convergence detection strategies (4000 steps, known region RMSE, and patch consistency). It appears that the Known Region RMSE method results in the top inpainting SSIM for the MCPN Emergent variant. This is likely to be caused by the optimisation dynamic that can be observed in the traces of Figure 6 (top row, blue lines), where the MCPN Emergent solution reaches a fairly stable plateau. This means that, on average, it may be more beneficial to train a bit longer to ensure all samples in the dataset can reach a stable plateau. The traces of the other two methods (top row, orange and green lines) do not exhibit this level of stability, as the Inpainting SSIM tends to decrease monotonically. This results in higher sensitivity in the number of optimisation steps and, in particular, the top inpainting SSIM is achieved early and consistently in fewer than 4000 steps. In contrast, the other convergence strategies prefer solutions after 4000 steps (as shown in Table 2) and they are likely to yield lower inpainting SSIM for MCPN Direct and Stacked baseline, as shown in Table 3.

Based on the above results, the rest of the experiments are conducted with the model-specific learning rates. To understand the intricacies of each synthesis method, results using all three convergence methods are obtained, and the optimal combination for each task is reported in the manuscript, while the complete records are presented in the Appendix A.

## 3. Evaluation

The proposed methods are evaluated on two common tasks related to remote sensing applications: (a) guided image inpainting and (b) guided super-resolution. Furthermore, to demonstrate their versatility, tests on image completion with other multi-modal image translation datasets are carried out.

### 3.1. Guided Satellite Image Inpainting

**Figure 7:**
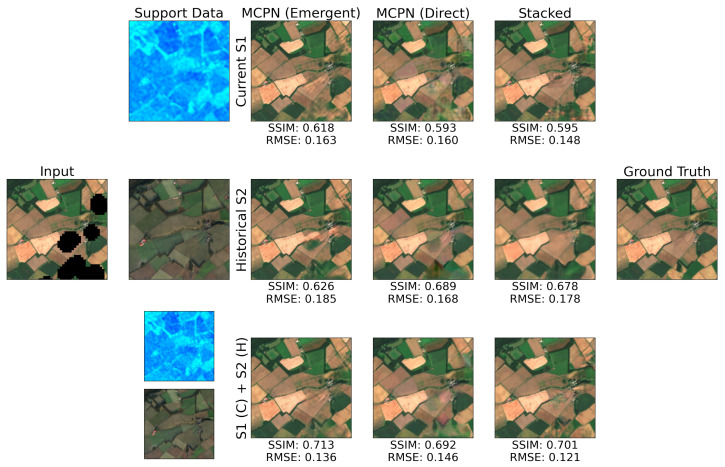
Comparison of reconstructed Sentinel-2 image for 3 types of support data (one per row) and the three tested methods.

In this work, the inpainting capability of MCPN is evaluated on Sentinel-2 optical data (Figure 7). Although this data source is employed extensively in Earth observations, the presence of atmospheric phenomena such as clouds usually limits its potential. To inform the reconstruction of occluded regions, temporally proximate data from the Sentinel-1 can be used, which is less affected by atmospheric conditions. However, the domain shift between the optical and radar sensing modalities is large. As an alternative, historical optical data could be used to inform the synthesis process about the fine structure of the scene. This naturally relies on the assumption that the structure of the scene has not changed significantly compared to the historic data acquisition. The dataset used in [16] and created using a framework described in [23], contains pairs of temporally proximate Sentinel-1 and Sentinel-2 images for a period of 2 years and has been employed to evaluate MCPN. More specifically, the clear sky images from Scotland in the year 2020 are used as targets for the inpainting task, and the clear sky images from 2019 are averaged and used as the historical informing prior. The dataset also contains realistic masks (extracted from cloud masks for dates when clouds where present), which are applied to clear sky images to measure the quality of synthesised samples with a precise ground truth.

In the scope of this work, the main comparison made in the context of inpainting is between the conventional stacked variant used in [16] and the proposed MCPN variants (Emergent and Direct). While other recent works on satellite image inpainting have been published, such as [24,25], they do not address the same problem treated in this work, as they do not operate on multi-modal guidance data and do not solve the problem via internal learning. Furthermore, at the time of writing, the source code and weights have not been provided for these works. The image inpainting task is also currently researched in the general domain of computer vision [26,27,28]; however, these solutions are not appropriate for the same reason as they focus on a single RGB modality and are pre-trained on datasets that are not relevant for the satellite image domain.

Similar to the experiments in Section 2.2, where convergence dynamics were studied, all models are optimised for 20,000 steps in order to compare several convergence detection methods. The obtained peak SSIM performance for the inpainted region, not practically achievable without access to the ground truth, is shown in Table 4. Although this is infeasible, it provides an indication of the upper bound performance of each method.

For the challenging case where Sentinel-1 is the informing signal, the Emergent variant of MCPN offers higher inpainted SSIM, compared to the other two methods. It can be observed that the whole image SSIM is drastically lower for the Stacked compared to MCPN Emergent. This is primarily caused by the fact that the extracted images yielding the maximised SSIM for the inpainting region are often premature in the case of the Stacked approach and the MCPN direct. For the historical Sentinel-2 case, the Stacked method achieves the highest SSIM, which could be attributed to the less severe domain shift between the informing and synthesised signals. For the case of combined Sentinel-1 and Sentinel-2 informing sources, the Emergent variant results in a higher SSIM for both the inpainted region and the whole image. In terms of peak performance, the direct variant of MCPN is not as performant as the other methods, which could potentially be attributed to the bottleneck aspect of the architecture. However, as described in Section 3.2, this MCPN variant is beneficial for the image super-resolution task.

In practice, one of the convergence detection methods described in Section 2.2 must be employed; namely, a constant number of steps, patch consistency metric, or RMSE of the known pixels. It has been found that the optimisation dynamics of each synthesis method are quite different, and hence, different convergence detection techniques are appropriate. Based on the results contained and discussed in the Appendix A, the Known Region RMSE metric works best for the Emergent variant of MCPN, while the constant of 4000 steps is most beneficial for the Direct MCPN and the baseline Stacked approach. Hence, the performances resulting from these choices are contained in Table 5, indicating the quality of synthesis that can be realistically achieved.

The highest quality of inpainting (as well as reconstruction of the known region) is achieved by employing the Emergent MCPN framework for both current Sentinel-1 and historical Sentinel-2 images. Furthermore, consistent with the earlier results in [16], the introduction of historical data from the same modality brings significantly higher benefits compared to the current cross-modal Sentinel-1 representation. The domain shift between the informing and synthesised signals (as in the case of Sentinel-1) remains difficult to handle for the convolutional parameterisation models. However, the use of the MCPN scheme offers a significant improvement of inpainting quality, where the inpainting SSIM of MCPN Emergent and Direct are 0.638 and 0.601, compared to 0.576, achieved by the Stacked approach.

It is important to note that the results are expected to have a high standard deviation when compared to the global mean. The reason for this is that the difficulty of the individual image tasks varies greatly, depending on how large the masked area is and how predictable the missing content is. Naturally, for some tasks, some of the missing information is not feasible to recover when appropriate priors are absent from the informing signal. For that reason, the discussion so far has focused on the mean performance value. A potential alternative is to apply a correcting factor per sample (with a sample being a fixed image and a fixed mask shape) that accounts for its difficulty. For each sample, the mean and standard deviation of the metric value across methods indicate the difficulty of that specific inpainting task. Based on the resulting sample-specific mean and standard deviation, a z-score can be computed for each method, providing a scaled measure of the difference in performance with respect to all methods. By averaging the z-score value across all samples, a dataset-wide z-score is achieved that corrects for the varying levels of difficulty. The resulting values are shown in Table 6, where a value of +0.900 indicates that, on average, a given method results in a metric value of 0.900 standard deviations higher than the mean for that sample. Furthermore, each value is accompanied by an indicator mark that corresponds to the result of a Wilcoxon signed-rank test [29] of a hypothesis that a given metric distribution is a significant improvement (*p* = 0.05) with respect to the baseline Stacked approach.

The average z-score values confirm the earlier conclusions, where the Emergent Core leads to a significant performance boost for the Sentinel-1 data mode. For the historical Sentinel-2 mode, the Stacked approach appears to work best; however, for the data mode containing both Sentinel-1 and Sentinel-2, the Emergent Core MCPN exhibits a very high quality of inpainting compared to the other two methods.

**Figure 8:**
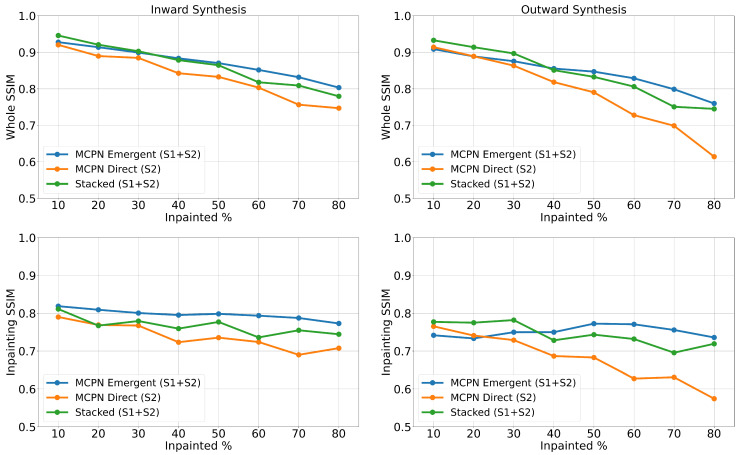
Traces indicating the changes of Structural Similarity Index (SSIM) values for different percentages of regions to inpaint.

To explore how the inpainting quality changes for different sizes of the synthesized region, a sweep of the mask size is conducted, where four clear-sky images from across the year are inpainted using a square mask with varying areas. Furthermore, both inward synthesis (where the synthesized region is fully surrounded by non-masked pixels) and outward synthesis (where the synthesized region is not surrounded by non-masked pixels) are explored by applying an inverse mask. The results of the sweep are shown in Figure 8, where the left column corresponds to inward synthesis, and the right column to outward synthesis. The two metrics of whole-image SSIM and inpainting SSIM are recorded for all three synthesis methods, each using the supporting data that provides the highest performance in Table 5 (S2 for MCPN Direct, and S1 + S2 for MCPN Emergent and Stacked).

The Emergent variant of MCPN (blue line) is leading significantly for all metrics if the inpainted region is 40% or more. The Direct variant of MCPN is outperformed by the Stacked method, which is consistent with the performance reported in Table 5 (0.692 Inpainting SSIM for MCPN Direct and 0.713 for Stacked).

### 3.2. Guided Satellite Image Super-Resolution

The multi-scale architectures can be readily adapted to perform a super-resolution task on the multi-modal representations. This can be achieved by employing a downsampling operation to the output of the target domain head and backpropagating gradients from a low-resolution source through it. Apart from this operation, the architecture of MCPN remains unchanged. Additional informing sources (such as the historical optical mean), inherently in high resolution, can be synthesised by the remaining domain heads along with the super-resolved image. This can help with producing structurally coherent upsampling. In all presented experimentation, the bilinear downsampling operation is selected. It is worth noting previous literature addressing a similar problem and commonly referring to it as guided super-resolution [30,31,32,33,34,35]. However, most of the previous works focus on the task of super-resolving a single-channel Depth image, based on a corresponding three-channel RGB image of higher resolution. This makes the application of many existing models to new problem settings difficult. Furthermore, MCPN constitutes a fully unsupervised framework, where no pretraining is carried out. This makes the PixTransform work introduced in [34] particularly appropriate as a baseline since it is also unsupervised. With minimal changes applied, to accommodate for three channels in the super-resolved image (rather than one, as in the depth image), it has been used for comparison in the conducted experiments. Furthermore, a common, externally-trained, baseline of EDSR [36] is tested as well (in this case, the low-resolution image is super-resolved without any guide image).

**Figure 9:**
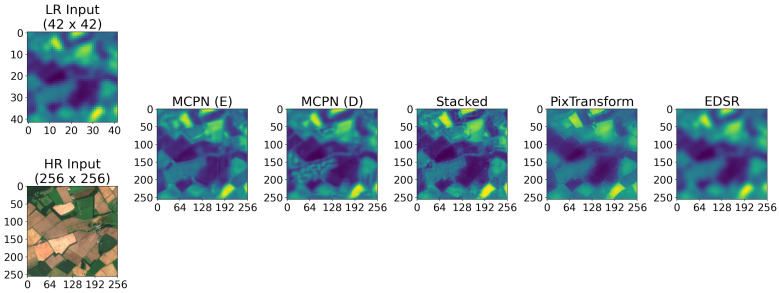
With the support of the image from the RGB bands (10 m resolution), the low-resolution SWIR image (Band 9 with 60 m resolution) is upsampled. This is an exploratory result since no ground truth exists for a high-resolution SWIR source.

With this adjustment, all three synthesis methods are employed to upsample an inherently low-resolution source. Here, Band 9 Sentinel-2 with SWIR data, with a resolution of 60 metres, is super-resolved by using the current RGB bands (Bands 4, 3, and 2) with 10 m resolution as the informing signal. The results are shown in Figure 9, along with the two employed baselines of PixTransform [34] and EDSR [36]. In this case, the target upscaling factor is close to 6, and for EDSR, it is achieved by passing the image through the model with factor 2 followed by the model with factor 4. The result of these two consecutive EDSR passes yields an 8 times larger image, which is then interpolated down to 256 × 256 px resolution with a bilinear operation. Similarly, since the PixTransform tool requires the upscaling factor to be a whole integer, the image is first upsampled to 64 × 64 pixels and then supplied as the low-resolution source. In the case of MCPN, any upscaling factor can be achieved by substituting an appropriate downsampling operation into the process, making it more flexible than the compared baselines. Since a high-resolution ground truth for Band 9 of Sentinel-2 does not exist, it is challenging to compare these results beyond visual impression. The EDSR method results in a significant perceived blur while the convolutional parameterisation methods increase the fidelity of the image. The output of the Stacked baseline and the Emergent Core MCPN appears to contain more fine details propagated from the RGB image compared to the Direct Core MCPN. The PixTransform output appears to produce high-quality fine details compared to the other methods, but it also appears to yield reduced contrast in some parts of the image.

**Figure 10:**
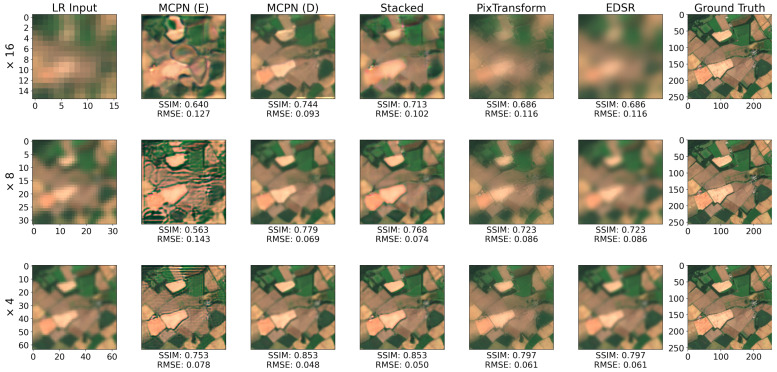
Example of super-resolution performance for several upscaling factors: ×16 (**top row**), ×8 (**middle row**), ×4 (**bottom row**). The informing high-resolution source used for the experiment was the historical optical mean from the previous year.

A quantitative evaluation approach is adopted here that relies on pairs of high-resolution images and their corresponding downsampled versions. The experiment has been conducted on 20 clear-sky RGB images from Scotland from the year 2020 with the supporting information of the historical average from the year 2019. In the process, each of the 20 images was subject to bilinear downsampling, which was subsequently used as the low-resolution input. The super-resolution performance achieved by each method is shown in Table 7, with the SSIM and RMSE values for three upscaling factors of 16, 8, and 4. Full results, for all convergence techniques and the ideal performance are included in Appendix A. It is shown that the MCPN Direct approach achieves superior performance for the task of super-resolution, consistently outperforming all other methods for all scaling factors. An example of the super-resolved outputs and a corresponding ground truth is shown in Figure 10, where MCPN Direct produces the highest quality output, especially for the larger factors, while MCPN Emergent introduces more artefacts to the super-resolved image since the correspondence between the two domain signals is not as constrained as in the case of the Direct Core variant. The PixTransform method appears to do well with reconstructing fine details in the scene (such as sharp region borders), but at the same time, it introduces a considerable color distortion, which ultimately leads to performance inferior to the MCPN Direct Core.

### 3.3. Guided Image Inpainting in Other Domains

The MCPN method can be applied to other tasks where spatially-aligned multi-modal data are available. Here, it is demonstrated how the same methods are used to perform image inpainting tasks on four common datasets that contain aligned multi-domain data: Facades, Maps, Night-to-Day, and CityScapes [8]. The task involves filling the square area in the middle of each image within a border of 50 pixels around the image periphery (this translates to about 37% of fill area for 256 × 256 px images).

The four datasets representing other types of tasks can be categorised into those containing a shallow descriptive guide, such as a segmentation mask (Facades, Maps, and CityScapes), and those containing a rich natural image guide (Night-to-Day). Depending on this aspect, a different convergence detection technique may be appropriate, and hence, the best performing one is applied on per-dataset and per-synthesis-method basis, as shown in Table 8 by indicating § as the 4000 steps technique, † as Known Region RMSE, and ‡ as Patch Consistency.

The results in Table 8 demonstrate that the MCPN variants can achieve superior performance to the Stacked baseline for all tasks (again, full results for all convergence detection methods and the ideal performance are included in Appendix A). For the datasets of Maps, Night-to-Day, and Cityscapes, the MCPN Emergent variant outperforms both other methods. For the task of inpainting Facade images based on a segmentation map, the Direct variant of MCPN exhibits higher performance than the Emergent variant. The Stacked baseline yields the lowest inpainting SSIM across all datasets, with the exception of the Night-to-Day, where it performs better than the Direct Core MCPN.

**Figure 11:**
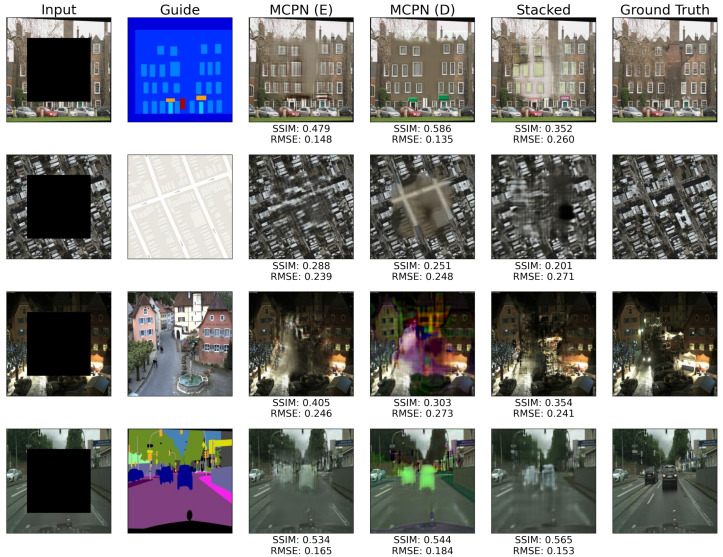
Example output for the three tested inpainting methods for each experiment dataset (from top to bottom: Facades, Map-to-Aerial, Night-to-Day, Cityscapes). The reported metric values correspond to the inpainted area only.

Some of the results for the above datasets are demonstrated in Figure 11. The tendency of the Stacked method to produce inpaintings inconsistent with the rest of the image is apparent, contributing to the highest errors associated with that technique. The Emergent Core MCPN produces higher structural distortions compared to the Direct Core MCPN.

## 4. Conclusions

The proposed method Multi-modal Convolutional Parameterisation Network (MCPN) demonstrates the capability of parameterising spatially aligned signals from multiple domains using convolutional neural network architectures. This capability enables an internal solution to several image inverse tasks, such as image completion or super-resolution. By definition, an MCPN model can readily be applied for any number of domains, and it has been shown that it can work with domain shifts as large as between image segmentation maps and corresponding natural images. The synthesis process takes in the order of several minutes on a single consumer-grade GPU and is fully unsupervised, requiring no external pretraining.

The Emergent Core variant of MCPN exhibits superior performance in the conducted experimentation for satellite image inpainting. This improvement is particularly significant for the difficult case of transferring information from the SAR source of Sentinel-1 to the optical image of Sentinel-2. Another use case for MCPN (including the direct Stacked approach) has been proposed, where an informing high-resolution image is used as a guide to super-resolve a spatially aligned low-resolution image. In this case, the MCPN Direct method exhibits the highest performance. This technique can potentially enable applications, where low-resolution remote sensing data sources are upsampled by transferring knowledge from other sensors to improve the fidelity of the observation. Finally, the evaluation contains results of image inpainting for other image datasets, where the MCPN methods provide a significant boost of the synthesis quality.

The established MCPN is a versatile tool that can facilitate internal learning of representations from disparate domains. The results indicate that this approach can significantly increase the quality of synthesis.

## Figures and Tables

**Table 1:** The key parameters of the Multi-Modal Convolutional Parameterisation Network (MCPN) model used to produce the results.

Parameter	Value
Core Network Base	[16, 32, 64, 128, 128, 128]
Core Network Skip	[4, 4, 4, 4, 4, 4]
Head Network Base	[32, 32]
Head Network Skip	[32, 32]
Head Kernel Size	3 × 3
Head Activation	None

**Table 2:** Optimal values of the ground truth reference and the two adaptive convergence methods along with the number of steps after which they were obtained.

Method	LR	Inpainting SSIM (GT) ↑	Known RMSE ↓	Patch Consistency ↓
	10^−4^	0.732 at 3600 ± 1579	0.022 at 19,325 ± 491	3.782 at 8600 ± 5839
MCPN Emergent	10^−3^	0.735 at 3725 ± 914	0.021 at 19,575 ± 449	3.805 at 15,275 ± 7553
	10^−2^	0.735 at 3575 ± 1380	0.021 at 19,525 ± 363	3.875 at 15,000 ± 7510
	10^−4^	0.706 at 800 ± 254	0.017 at 19,400 ± 494	2.989 at 5375 ± 1028
MCPN Direct	10^−3^	0.689 at 1350 ± 390	0.018 at 19,600 ± 212	3.322 at 6425 ± 1987
	10^−2^	0.714 at 1000 ± 158	0.017 at 19,850 ± 50	3.025 at 5750 ± 1425
	10^−4^	0.713 at 75 ± 43	0.011 at 17,650 ± 3332	2.261 at 9700 ± 5980
Stacked	10^−3^	0.712 at 1400 ± 2136	0.011 at 19,450 ± 384	2.032 at 9025 ± 4935
	10^−2^	0.716 at 3450 ± 3377	0.011 at 16,525 ± 5063	2.367 at 8675 ± 4246

**Table 3:** Mean inpainting Structural Similarity Index (SSIM) values achieved for 4 repetitions carried out on the entire Scotland dataset (Synthetic Aperture Radar (SAR)-to-optical translation).

Method	LR	Ideal (GT)	4000 Steps	Known RMSE	Patch Consistency
MCPN Emergent	10^−3^	0.677 ± 0.071	0.626 ± 0.160	0.669 ± 0.066	0.637 ± 0.098
MCPN Direct	10^−2^	0.663 ± 0.069	0.611 ± 0.070	0.521 ± 0.080	0.601 ± 0.074
Stacked	10^−2^	0.650 ± 0.079	0.573 ± 0.090	0.545 ± 0.078	0.570 ± 0.087

**Table 4:** Results for the Scotland dataset: peak performance.

Guidance			MCPN (Emergent Core)	MCPN (Direct Core)	Stacked
Current Sentinel-1	Whole	SSIM ↑	0.854 ± 0.041	0.760 ± 0.052	0.743 ± 0.053
		RMSE ↓	0.079 ± 0.053	0.088 ± 0.029	0.092 ± 0.041
	Inpainting	SSIM ↑	0.665 ± 0.082	0.657 ± 0.072	0.661 ± 0.077
		RMSE ↓	0.137 ± 0.090	0.130 ± 0.053	0.131 ± 0.063
Historical Sentinel-2	Whole	SSIM ↑	0.879 ± 0.044	0.853 ± 0.069	0.879 ± 0.062
		RMSE ↓	0.081 ± 0.062	0.072 ± 0.026	0.071 ± 0.046
	Inpainting	SSIM ↑	0.719 ± 0.113	0.714 ± 0.068	0.738 ± 0.090
		RMSE ↓	0.142 ± 0.108	0.120 ± 0.039	0.120 ± 0.069
Current Sentinel-1 + Historical Sentinel-2	Whole	SSIM ↑	0.876 ± 0.036	0.838 ± 0.056	0.869 ± 0.065
		RMSE ↓	0.071 ± 0.055	0.071 ± 0.030	0.066 ± 0.038
	Inpainting	SSIM ↑	0.743 ± 0.098	0.694 ± 0.064	0.741 ± 0.083
		RMSE ↓	0.121 ± 0.101	0.117 ± 0.050	0.111 ± 0.059

**Table 5:** Results for the Scotland dataset: achievable performance with optimal convergence detection.

Guidance			MCPN (Emergent Core)	MCPN (Direct Core)	Stacked
			Known RMSE	4000 Steps	4000 Steps
Current Sentinel-1	Whole	SSIM ↑	0.859 ± 0.041	0.824 ± 0.045	0.837 ± 0.063
		RMSE ↓	0.079 ± 0.048	0.081 ± 0.033	0.086 ± 0.050
	Inpainting	SSIM ↑	0.638 ± 0.081	0.601 ± 0.068	0.576 ± 0.080
		RMSE ↓	0.141 ± 0.082	0.140 ± 0.055	0.149 ± 0.071
Historical Sentinel-2	Whole	SSIM ↑	0.880 ± 0.043	0.864 ± 0.041	0.875 ± 0.063
		RMSE ↓	0.079 ± 0.051	0.075 ± 0.028	0.086 ± 0.059
	Inpainting	SSIM ↑	0.698 ± 0.107	0.692 ± 0.075	0.703 ± 0.105
		RMSE ↓	0.142 ± 0.089	0.131 ± 0.044	0.147 ± 0.089
Current Sentinel-1 + Historical Sentinel-2	Whole	SSIM ↑	0.882 ± 0.036	0.861 ± 0.039	0.879 ± 0.055
		RMSE ↓	0.069 ± 0.048	0.071 ± 0.032	0.074 ± 0.049
	Inpainting	SSIM ↑	0.735 ± 0.096	0.679 ± 0.070	0.713 ± 0.100
		RMSE ↓	0.120 ± 0.087	0.124 ± 0.054	0.128 ± 0.076

**Table 6:** Results for the Scotland dataset: achievable average difference from mean performance (numbers correspond to multiples of standard deviations).

Guidance			MCPN (Emergent Core)	MCPN (Direct Core)	Stacked
			Known RMSE	4000 Steps	4000 Steps
Current Sentinel-1	Whole	SSIM ↑	+0.900 ✓	−0.993 ✗	+0.094
		RMSE ↓	−0.555 ✓	+0.417 ✗	+0.138
	Inpainting	SSIM ↑	+0.900 ✓	−0.168 ✓	−0.732
		RMSE ↓	−0.488 ✓	+0.175 ✓	+0.313
Historical Sentinel-2	Whole	SSIM ↑	+0.207 ✗	−0.652 ✗	+0.445
		RMSE ↓	−0.100 ✓	+0.139 ✗	−0.039
	Inpainting	SSIM ↑	−0.031 ✗	−0.212 ✗	+0.243
		RMSE ↓	−0.032 ✗	+0.056 ✓	−0.024
Current Sentinel-1 + Historical Sentinel-2	Whole	SSIM ↑	+0.405 ✗	−0.953 ✗	+0.548
		RMSE ↓	−0.452 ✓	+0.526 ✗	−0.074
	Inpainting	SSIM ↑	+0.738 ✓	−0.823 ✗	+0.085
		RMSE ↓	−0.521 ✓	+0.489 ✗	+0.032

**Table 7:** Results for the super-resolution task (historical high-resolution optical reference used for super-resolving current downsampled optical (achievable performance).

Factor		MCPN (Emergent Core)	MCPN (Direct Core)	Stacked	PixTransform [34]	EDSR [36]
		4000 Steps	Known RMSE	4000 Steps		
×16	SSIM ↑	0.487 ± 0.137	0.733 ± 0.068	0.719 ± 0.072	0.718 ± 0.060	0.699 ± 0.055
	RMSE ↓	0.184 ± 0.057	0.085 ± 0.047	0.094 ± 0.060	0.094 ± 0.046	0.098 ± 0.045
×8	SSIM ↑	0.584 ± 0.168	0.782 ± 0.049	0.771 ± 0.085	0.758 ± 0.052	0.727 ± 0.050
	RMSE ↓	0.135 ± 0.067	0.064 ± 0.029	0.076 ± 0.061	0.076 ± 0.039	0.080 ± 0.040
×4	SSIM ↑	0.685 ± 0.137	0.847 ± 0.029	0.825 ± 0.084	0.815 ± 0.044	0.789 ± 0.038
	RMSE ↓	0.104 ± 0.068	0.047 ± 0.017	0.062 ± 0.057	0.061 ± 0.031	0.061 ± 0.031

**Table 8:** Inpainting results for the four spatially-aligned multi-domain datasets. Mean values along with corresponding standard deviations are reported. Metrics for both whole image comparison (Whole) and inpainting comparison (Inpainting) are shown. (Achievable Performance). §—4000 steps, †—known reconstruction root mean square error, ‡—patch consistency.

Guidance			MCPN (Emergent Core)	MCPN (Direct Core)	Stacked
Facades (Segmentation → Building)	Whole	SSIM ↑	0.763 ± 0.044 §	0.784 ± 0.058 †	0.720 ± 0.100 †
		RMSE ↓	0.108 ± 0.031 §	0.113 ± 0.040 †	0.118 ± 0.038 †
	Inpainting	SSIM ↑	0.476 ± 0.109 §	0.505 ± 0.144 †	0.453 ± 0.130 †
		RMSE ↓	0.172 ± 0.051 §	0.183 ± 0.067 †	0.184 ± 0.061 †
Maps (Map → Aerial)	Whole	SSIM ↑	0.791 ± 0.070 †	0.768 ± 0.074 §	0.744 ± 0.076 §
		RMSE ↓	0.085 ± 0.031 †	0.085 ± 0.030 §	0.113 ± 0.038 §
	Inpainting	SSIM ↑	0.512 ± 0.174 †	0.510 ± 0.175 §	0.404 ± 0.169 §
		RMSE ↓	0.137 ± 0.051 †	0.134 ± 0.050 §	0.183 ± 0.061 §
Night-to-Day (Day → Night)	Whole	SSIM ↑	0.870 ± 0.068 †	0.769 ± 0.093 ‡	0.828 ± 0.103 ‡
		RMSE ↓	0.075 ± 0.041 †	0.100 ± 0.038 ‡	0.096 ± 0.055 ‡
	Inpainting	SSIM ↑	0.709 ± 0.167 †	0.576 ± 0.148 ‡	0.644 ± 0.178 ‡
		RMSE ↓	0.121 ± 0.067 †	0.157 ± 0.060 ‡	0.150 ± 0.076 ‡
Cityscapes (Segmentation → Street)	Whole	SSIM ↑	0.822 ± 0.031 §	0.793 ± 0.041 §	0.802 ± 0.047 §
		RMSE ↓	0.093 ± 0.030 §	0.092 ± 0.023 §	0.093 ± 0.028 §
	Inpainting	SSIM ↑	0.613 ± 0.077 §	0.610 ± 0.071 §	0.608 ± 0.077 §
		RMSE ↓	0.150 ± 0.050 §	0.143 ± 0.037 §	0.147 ± 0.047 §

## Data Availability

The dataset comprises two-year temporal coverage measurements for two different regions, one in Scotland the other in India, each containing approximately 200 samples. The data are available at https://doi.org/10.5281/zenodo.5897695 (accessed on 13 December 2023).

## Abbreviations

The following abbreviations are used in this manuscript:

MCPN	Multi-modal Convolutional Parameterisation Network
SAR	Synthetic Aperture Radar
DIP	Deep Image Prior

## Appendix A. Convergence Detection

The complete record of the results using individual convergence detection methods is provided here for all three tasks (satellite image inpainting, super-resolution, and inpainting in additional visual domains).

### Appendix A.1. Satellite Inpainting

For the MCPN Emergent, both patch consistency and 4000 step methods (Table A1 and Table A2, respectively) are outperfomed by the known reconstruction RMSE (Table A3). In the case of MCPN Direct, the 4000 step results in the highest value of Inpainting SSIM, at the cost of a slightly lower Whole Image SSIM. Since the quality of the inpainted region is the priority in this work, the 4000 step method is used for the MCPN Direct variant. Finally, for the Stacked baseline, the 4000 step method also appears to be most beneficial for the inpainting quality (however, patch consistency comes close). Again, this appears to come at the cost of the lower value of SSIM of the entire image. The likely reason for this is that the highest SSIM of inpainting is achieved early in the process before the high-resolution detail of the image is fit by the network, which agrees with the traces shown in Figure 6. This effect is not apparent for the proposed MCPN Emergent variant, making it a good choice for a stable synthesis framework.

**Table A1:** Satellite inpainting results for the Scotland dataset. Metrics for both whole image comparison (Whole) and inpainting comparison (Inpainting) are shown (4000 steps).

Guidance			MCPN (Emergent Core)	MCPN (Direct Core)	Stacked
Current Sentinel-1	Whole	SSIM ↑	0.833 ± 0.065	0.824 ± 0.045	0.837 ± 0.063
		RMSE ↓	0.113 ± 0.104	0.081 ± 0.033	0.086 ± 0.050
	Inpainting	SSIM ↑	0.604 ± 0.170	0.601 ± 0.068	0.576 ± 0.080
		RMSE ↓	0.203 ± 0.186	0.140 ± 0.055	0.149 ± 0.071
Historical Sentinel-2	Whole	SSIM ↑	0.857 ± 0.070	0.864 ± 0.041	0.875 ± 0.063
		RMSE ↓	0.119 ± 0.106	0.075 ± 0.028	0.086 ± 0.059
	Inpainting	SSIM ↑	0.654 ± 0.191	0.692 ± 0.075	0.703 ± 0.105
		RMSE ↓	0.217 ± 0.190	0.131 ± 0.044	0.147 ± 0.089
Current Sentinel-1 + Historical Sentinel-2	Whole	SSIM ↑	0.855 ± 0.060	0.861 ± 0.039	0.879 ± 0.055
		RMSE ↓	0.091 ± 0.095	0.071 ± 0.032	0.074 ± 0.049
	Inpainting	SSIM ↑	0.700 ± 0.169	0.679 ± 0.070	0.713 ± 0.100
		RMSE ↓	0.158 ± 0.170	0.124 ± 0.054	0.128 ± 0.076

**Table A2:** Satellite inpainting results for the Scotland dataset. Metrics for both whole image comparison (Whole) and inpainting comparison (Inpainting) are shown (patch consistency).

Guidance			MCPN (Emergent Core)	MCPN (Direct Core)	Stacked
Current Sentinel-1	Whole	SSIM ↑	0.850 ± 0.051	0.830 ± 0.050	0.839 ± 0.059
		RMSE ↓	0.089 ± 0.071	0.081 ± 0.032	0.084 ± 0.047
	Inpainting	SSIM ↑	0.621 ± 0.108	0.588 ± 0.069	0.564 ± 0.082
		RMSE ↓	0.159 ± 0.124	0.141 ± 0.053	0.149 ± 0.077
Historical Sentinel-2	Whole	SSIM ↑	0.874 ± 0.054	0.872 ± 0.052	0.887 ± 0.050
		RMSE ↓	0.089 ± 0.077	0.076 ± 0.031	0.079 ± 0.055
	Inpainting	SSIM ↑	0.689 ± 0.135	0.690 ± 0.082	0.706 ± 0.105
		RMSE ↓	0.158 ± 0.133	0.133 ± 0.046	0.139 ± 0.093
Current Sentinel-1 + Historical Sentinel-2	Whole	SSIM ↑	0.873 ± 0.048	0.867 ± 0.053	0.887 ± 0.047
		RMSE ↓	0.077 ± 0.069	0.073 ± 0.036	0.070 ± 0.044
	Inpainting	SSIM ↑	0.721 ± 0.122	0.669 ± 0.071	0.713 ± 0.093
		RMSE ↓	0.134 ± 0.120	0.128 ± 0.054	0.123 ± 0.071

**Table A3:** Satellite inpainting results for the Scotland dataset. Metrics for both whole image comparison (Whole) and inpainting comparison (Inpainting) are shown (known root mean square error).

Guidance			MCPN (Emergent Core)	MCPN (Direct Core)	Stacked
Current Sentinel-1	Whole	SSIM ↑	0.859 ± 0.041	0.834 ± 0.061	0.849 ± 0.060
		RMSE ↓	0.079 ± 0.048	0.086 ± 0.034	0.080 ± 0.045
	Inpainting	SSIM ↑	0.638 ± 0.081	0.532 ± 0.071	0.538 ± 0.079
		RMSE ↓	0.141 ± 0.082	0.153 ± 0.050	0.146 ± 0.073
Historical Sentinel-2	Whole	SSIM ↑	0.880 ± 0.043	0.885 ± 0.048	0.896 ± 0.048
		RMSE ↓	0.079 ± 0.051	0.080 ± 0.029	0.075 ± 0.050
	Inpainting	SSIM ↑	0.698 ± 0.107	0.670 ± 0.079	0.701 ± 0.094
		RMSE ↓	0.142 ± 0.089	0.143 ± 0.040	0.133 ± 0.080
Current Sentinel-1 + Historical Sentinel-2	Whole	SSIM ↑	0.882 ± 0.036	0.882 ± 0.045	0.896 ± 0.046
		RMSE ↓	0.069 ± 0.048	0.073 ± 0.031	0.068 ± 0.042
	Inpainting	SSIM ↑	0.735 ± 0.096	0.654 ± 0.064	0.703 ± 0.091
		RMSE ↓	0.120 ± 0.087	0.132 ± 0.049	0.122 ± 0.067

### Appendix A.2. Guided Super-Resolution

For the MCPN Emergent variant, it appears that the 4000 step convergence is most beneficial on the super-resolution task, leading to a considerably higher value of SSIM for big scaling factors, such as 16. For the smaller scaling factor of 4, the patch consistency convergence detection yields a higher result, but 4000 steps are still selected as the preferred method for MCPN Emergent super-resolution since it handles big scaling factors significantly better. For the MCPN Direct variant, the known reconstruction RMSE yields superior performance to both other methods and across all scaling factors and is hence used for that synthesis model. For the Stacked baseline, stopping after 4000 steps provides better quality of super-resolution for large scaling factors and is therefore used as the convergence detection for that method. Table A4, Table A5, Table A6 and Table A7 contain full results for the ideal performance and all three convergence methods.

**Table A4:** Results for the guided super-resolution task (historical high-resolution optical reference used for super-resolving current downsampled optical. Metrics for both whole image comparison (Whole) and inpainting comparison (Inpainting) are shown (ideal performance).

Factor		MCPN (Emergent Core)	MCPN (Direct Core)	Stacked	PixTransform [34]	EDSR
×16	SSIM ↑	0.641 ± 0.090	0.735 ± 0.068	0.731 ± 0.066	0.718 ± 0.060	0.699 ± 0.055
	RMSE ↓	0.140 ± 0.064	0.085 ± 0.047	0.090 ± 0.052	0.094 ± 0.046	0.098 ± 0.045
×8	SSIM ↑	0.716 ± 0.088	0.782 ± 0.049	0.783 ± 0.072	0.758 ± 0.052	0.727 ± 0.050
	RMSE ↓	0.098 ± 0.057	0.064 ± 0.029	0.071 ± 0.050	0.076 ± 0.039	0.080 ± 0.040
×4	SSIM ↑	0.783 ± 0.085	0.848 ± 0.029	0.840 ± 0.070	0.815 ± 0.044	0.789 ± 0.038
	RMSE ↓	0.075 ± 0.047	0.047 ± 0.017	0.055 ± 0.044	0.061 ± 0.031	0.061 ± 0.031

**Table A5:** Results for the Guided Super-Resolution Task (Historical High-Resolution Optical Reference Used for Super-Resolving Current Downsampled Optical. Metrics for both whole image comparison (Whole) and inpainting comparison (Inpainting) are shown. (4000 steps).

Factor		MCPN (Emergent Core)	MCPN (Direct Core)	Stacked	PixTransform [34]	EDSR
×16	SSIM ↑	0.487 ± 0.137	0.710 ± 0.062	0.719 ± 0.072	0.718 ± 0.060	0.699 ± 0.055
	RMSE ↓	0.184 ± 0.057	0.091 ± 0.047	0.094 ± 0.060	0.094 ± 0.046	0.098 ± 0.045
×8	SSIM ↑	0.584 ± 0.168	0.748 ± 0.047	0.771 ± 0.085	0.758 ± 0.052	0.727 ± 0.050
	RMSE ↓	0.135 ± 0.067	0.071 ± 0.032	0.076 ± 0.061	0.076 ± 0.039	0.080 ± 0.040
×4	SSIM ↑	0.685 ± 0.137	0.809 ± 0.031	0.825 ± 0.084	0.815 ± 0.044	0.789 ± 0.038
	RMSE ↓	0.104 ± 0.068	0.054 ± 0.019	0.062 ± 0.057	0.061 ± 0.031	0.061 ± 0.031

**Table A6:** Results for the guided super-resolution task (historical high-resolution optical reference used for super-resolving current downsampled optical. Metrics for both whole image comparison (Whole) and inpainting comparison (Inpainting) are shown (patch consistency).

Factor		MCPN (Emergent Core)	MCPN (Direct Core)	Stacked	PixTransform [34]	EDSR
×16	SSIM ↑	0.410 ± 0.169	0.728 ± 0.068	0.695 ± 0.078	0.718 ± 0.060	0.699 ± 0.055
	RMSE ↓	0.212 ± 0.075	0.087 ± 0.047	0.102 ± 0.071	0.094 ± 0.046	0.098 ± 0.045
×8	SSIM ↑	0.605 ± 0.137	0.780 ± 0.049	0.758 ± 0.086	0.758 ± 0.052	0.727 ± 0.050
	RMSE ↓	0.131 ± 0.072	0.065 ± 0.029	0.080 ± 0.065	0.076 ± 0.039	0.080 ± 0.040
×4	SSIM ↑	0.720 ± 0.160	0.845 ± 0.030	0.830 ± 0.091	0.815 ± 0.044	0.789 ± 0.038
	RMSE ↓	0.104 ± 0.115	0.047 ± 0.017	0.059 ± 0.053	0.061 ± 0.031	0.061 ± 0.031

**Table A7:** Results for the guided super-resolution task (historical high-resolution optical reference used for super-resolving current downsampled optical. Metrics for both whole image comparison (Whole) and inpainting comparison (Inpainting) are shown (known root mean square error).

Factor		MCPN (Emergent Core)	MCPN (Direct Core)	Stacked	PixTransform [34]	EDSR
×16	SSIM ↑	0.389 ± 0.180	0.733 ± 0.068	0.695 ± 0.078	0.718 ± 0.060	0.699 ± 0.055
	RMSE ↓	0.213 ± 0.081	0.085 ± 0.047	0.102 ± 0.071	0.094 ± 0.046	0.098 ± 0.045
×8	SSIM ↑	0.541 ± 0.201	0.782 ± 0.049	0.759 ± 0.089	0.758 ± 0.052	0.727 ± 0.050
	RMSE ↓	0.150 ± 0.081	0.064 ± 0.029	0.081 ± 0.070	0.076 ± 0.039	0.080 ± 0.040
×4	SSIM ↑	0.674 ± 0.154	0.847 ± 0.029	0.829 ± 0.096	0.815 ± 0.044	0.789 ± 0.038
	RMSE ↓	0.100 ± 0.068	0.047 ± 0.017	0.060 ± 0.056	0.061 ± 0.031	0.061 ± 0.031

### Appendix A.3. Other Tasks

The optimal convergence detection method has been found to vary depending on the type of synthesis method as well as the specific dataset task, as shown in Table A8. Table A9, Table A10 and Table A11 contain full results for each convergence detection method.

**Table A8:** Inpainting results for the four spatially-aligned multi-domain datasets. Mean values along with corresponding standard deviations are reported. Metrics for both whole image comparison (Whole) and inpainting comparison (Inpainting) are shown (ideal performance).

Guidance			MCPN (Emergent Core)	MCPN (Direct Core)	Stacked
Facades (Segmentation → Building)	Whole	SSIM ↑	0.754 ± 0.062	0.761 ± 0.100	0.702 ± 0.115
		RMSE ↓	0.107 ± 0.030	0.106 ± 0.034	0.109 ± 0.035
	Inpainting	SSIM ↑	0.496 ± 0.104	0.532 ± 0.128	0.532 ± 0.123
		RMSE ↓	0.169 ± 0.050	0.166 ± 0.050	0.158 ± 0.049
Maps (Map → Aerial)	Whole	SSIM ↑	0.792 ± 0.069	0.733 ± 0.118	0.607 ± 0.152
		RMSE ↓	0.082 ± 0.031	0.084 ± 0.030	0.111 ± 0.041
	Inpainting	SSIM ↑	0.533 ± 0.173	0.540 ± 0.168	0.499 ± 0.184
		RMSE ↓	0.131 ± 0.051	0.124 ± 0.047	0.147 ± 0.062
Night-to-Day (Day → Night)	Whole	SSIM ↑	0.868 ± 0.076	0.753 ± 0.116	0.807 ± 0.115
		RMSE ↓	0.072 ± 0.040	0.094 ± 0.035	0.081 ± 0.042
	Inpainting	SSIM ↑	0.727 ± 0.161	0.668 ± 0.141	0.729 ± 0.158
		RMSE ↓	0.114 ± 0.064	0.129 ± 0.053	0.112 ± 0.065
Cityscapes (Segmentation → Street)	Whole	SSIM ↑	0.830 ± 0.031	0.743 ± 0.050	0.747 ± 0.055
		RMSE ↓	0.086 ± 0.028	0.088 ± 0.018	0.083 ± 0.019
	Inpainting	SSIM ↑	0.625 ± 0.075	0.649 ± 0.066	0.672 ± 0.065
		RMSE ↓	0.138 ± 0.046	0.120 ± 0.029	0.110 ± 0.030

**Table A9:** Inpainting results for the four spatially-aligned multi-domain datasets. Mean values along with corresponding standard deviations are reported. Metrics for both whole image comparison (Whole) and inpainting comparison (Inpainting) are shown (4000 steps).

Guidance			MCPN (Emergent Core)	MCPN (Direct Core)	Stacked
Facades (Segmentation → Building)	Whole	SSIM ↑	0.763 ± 0.044	0.700 ± 0.076	0.720 ± 0.100
		RMSE ↓	0.108 ± 0.031	0.129 ± 0.035	0.118 ± 0.038
	Inpainting	SSIM ↑	0.476 ± 0.109	0.447 ± 0.132	0.453 ± 0.130
		RMSE ↓	0.172 ± 0.051	0.200 ± 0.058	0.184 ± 0.061
Maps (Map → Aerial)	Whole	SSIM ↑	0.759 ± 0.069	0.768 ± 0.074	0.744 ± 0.076
		RMSE ↓	0.112 ± 0.056	0.085 ± 0.030	0.113 ± 0.038
	Inpainting	SSIM ↑	0.472 ± 0.167	0.510 ± 0.175	0.404 ± 0.169
		RMSE ↓	0.180 ± 0.093	0.134 ± 0.050	0.183 ± 0.061
Night-to-Day (Day → Night)	Whole	SSIM ↑	0.804 ± 0.103	0.767 ± 0.092	0.823 ± 0.082
		RMSE ↓	0.157 ± 0.124	0.103 ± 0.038	0.113 ± 0.060
	Inpainting	SSIM ↑	0.570 ± 0.245	0.552 ± 0.171	0.605 ± 0.199
		RMSE ↓	0.254 ± 0.205	0.164 ± 0.063	0.183 ± 0.100
Cityscapes (Segmentation → Street)	Whole	SSIM ↑	0.822 ± 0.031	0.793 ± 0.041	0.802 ± 0.047
		RMSE ↓	0.093 ± 0.030	0.092 ± 0.023	0.093 ± 0.028
	Inpainting	SSIM ↑	0.613 ± 0.077	0.610 ± 0.071	0.608 ± 0.077
		RMSE ↓	0.150 ± 0.050	0.143 ± 0.037	0.147 ± 0.047

**Table A10:** Inpainting results for the four spatially-aligned multi-domain datasets. Mean values along with corresponding standard deviations are reported. Metrics for both whole image comparison (Whole) and inpainting comparison (Inpainting) are shown (known root mean square error).

Guidance			MCPN (Emergent Core)	MCPN (Direct Core)	Stacked
Facades (Segmentation → Building)	Whole	SSIM ↑	0.764 ± 0.043	0.784 ± 0.058	0.771 ± 0.058
		RMSE ↓	0.112 ± 0.029	0.113 ± 0.040	0.110 ± 0.037
	Inpainting	SSIM ↑	0.446 ± 0.106	0.505 ± 0.144	0.437 ± 0.145
		RMSE ↓	0.182 ± 0.048	0.183 ± 0.067	0.180 ± 0.061
Maps (Map → Aerial)	Whole	SSIM ↑	0.791 ± 0.070	0.798 ± 0.069	0.756 ± 0.077
		RMSE ↓	0.085 ± 0.031	0.082 ± 0.029	0.098 ± 0.036
	Inpainting	SSIM ↑	0.512 ± 0.174	0.505 ± 0.172	0.389 ± 0.195
		RMSE ↓	0.137 ± 0.051	0.134 ± 0.048	0.160 ± 0.059
Night-to-Day (Day → Night)	Whole	SSIM ↑	0.870 ± 0.068	0.751 ± 0.080	0.842 ± 0.079
		RMSE ↓	0.075 ± 0.041	0.121 ± 0.040	0.097 ± 0.057
	Inpainting	SSIM ↑	0.709 ± 0.167	0.464 ± 0.159	0.620 ± 0.197
		RMSE ↓	0.121 ± 0.067	0.196 ± 0.066	0.159 ± 0.094
Cityscapes (Segmentation → Street)	Whole	SSIM ↑	0.831 ± 0.030	0.821 ± 0.029	0.825 ± 0.028
		RMSE ↓	0.088 ± 0.027	0.094 ± 0.021	0.084 ± 0.020
	Inpainting	SSIM ↑	0.604 ± 0.076	0.593 ± 0.069	0.569 ± 0.071
		RMSE ↓	0.143 ± 0.045	0.153 ± 0.035	0.138 ± 0.033

**Table A11:** Inpainting results for the four spatially-aligned multi-domain datasets. Mean values along with corresponding standard deviations are reported. Metrics for both whole image comparison (Whole) and inpainting comparison (Inpainting) are shown (patch consistency).

Guidance			MCPN (Emergent Core)	MCPN (Direct Core)	Stacked
Facades (Segmentation → Building)	Whole	SSIM ↑	0.759 ± 0.046	0.734 ± 0.091	0.740 ± 0.089
		RMSE ↓	0.113 ± 0.029	0.121 ± 0.039	0.122 ± 0.042
	Inpainting	SSIM ↑	0.450 ± 0.103	0.478 ± 0.138	0.442 ± 0.138
		RMSE ↓	0.182 ± 0.048	0.189 ± 0.061	0.194 ± 0.065
Maps (Map → Aerial)	Whole	SSIM ↑	0.774 ± 0.064	0.771 ± 0.088	0.751 ± 0.074
		RMSE ↓	0.102 ± 0.060	0.086 ± 0.030	0.101 ± 0.035
	Inpainting	SSIM ↑	0.478 ± 0.161	0.506 ± 0.170	0.392 ± 0.180
		RMSE ↓	0.164 ± 0.099	0.134 ± 0.048	0.164 ± 0.058
Night-to-Day (Day → Night)	Whole	SSIM ↑	0.851 ± 0.069	0.769 ± 0.093	0.828 ± 0.103
		RMSE ↓	0.085 ± 0.042	0.100 ± 0.038	0.096 ± 0.055
	Inpainting	SSIM ↑	0.672 ± 0.166	0.576 ± 0.148	0.644 ± 0.178
		RMSE ↓	0.137 ± 0.069	0.157 ± 0.060	0.150 ± 0.076
Cityscapes (Segmentation → Street)	Whole	SSIM ↑	0.825 ± 0.028	0.801 ± 0.034	0.820 ± 0.032
		RMSE ↓	0.094 ± 0.030	0.090 ± 0.020	0.093 ± 0.026
	Inpainting	SSIM ↑	0.598 ± 0.069	0.609 ± 0.067	0.592 ± 0.067
		RMSE ↓	0.153 ± 0.050	0.142 ± 0.034	0.150 ± 0.042
